# Effects of Safe Early Intervention Approach: A 3‐Year Follow‐Up

**DOI:** 10.1002/brb3.71675

**Published:** 2026-08-17

**Authors:** Bulent Elbasan, Ecem Yildiz Cangur, Pelin Atalan Efkere, Kivilcim Gucuyener

**Affiliations:** ^1^ Department of Physiotherapy and Rehabilitation Faculty of Health Sciences Gazi University Ankara Turkey; ^2^ Private Clinic Ankara Turkey

**Keywords:** early intervention, infants at risk, motor development, SAFE, sensory development

## Abstract

**Purpose:**

Early intervention improves developmental outcomes in infants at risk. The SAFE early intervention approach has been found to be effective across multiple developmental domains. The aim of this study was to comparatively report the 3‐year follow‐up outcomes of the SAFE early intervention approach.

**Methods:**

The study included children aged 2 and 3 years who had been identified as being at neurodevelopmental risk during early infancy and had received either the SAFE early intervention approach or a Neurodevelopmental Treatment (NDT)‐based intervention. This follow‐up study was based on a doctoral dissertation that originally enrolled 41 infants. At follow‐up, 22 children were assessed at 2 years of age (13 SAFE, 9 NDT) and 19 children at 3 years of age (11 SAFE, 8 NDT). Development was evaluated using the Bayley Scales of Infant and Toddler Development, Third Edition (Bayley‐III), and sensory processing was assessed using Dunn's Sensory Profile (7–36 months). Longitudinal changes in Bayley‐III composite scores were analyzed using two‐way repeated‐measures analysis of variance following multiple imputation for missing data, while sensory processing outcomes were compared cross‐sectionally.

**Results:**

Cognitive and language composite scores improved significantly from 2 to 3 years of age in both the SAFE and NDT groups (*p* < 0.05), whereas no significant change was observed in motor composite scores. No significant between‐group differences were identified in cognitive, language, or motor composite scores at either assessment point, and no significant group × time interactions were found for any Bayley‐III composite score (*p* > 0.05). Cross‐sectional analyses of sensory processing revealed significant differences in selected domains in favor of the SAFE group at both 2 and 3 years of age (*p* < 0.05).

**Conclusion:**

Both the SAFE and NDT approaches supported developmental progress over time. Although no differences were observed in developmental trajectories between the groups, the SAFE approach showed potential advantages in selected aspects of sensory processing. Further studies with larger samples and longer follow‐up periods are needed to confirm these findings.

## Graphical abstract

What's Known?

- Early intervention can prevent developmental delays.
- The SAFE early intervention approach implemented during the first months of life has positive effects across developmental domains.

What's new?

- This study is the first to report long‐term follow‐up outcomes of the SAFE early intervention approach.
- Although developmental trajectories were similar between the SAFE and NDT groups, children who received the SAFE approach demonstrated advantages in selected aspects of sensory processing during follow‐up.

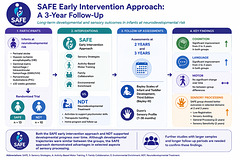

**Abbreviations**
BAYLEY‐III: Bayley Scales of Infant and Toddler Development, Third Edition
CP: Cerebral Palsy
CS: Cesarean section
NDT: Neurodevelopmental Treatment
SAFE: S—Sensory Strategies, A—Activity‐Based Motor Training, F—Family Collaboration, E—Environmental Enrichment
SPSS: Statistical Package for the Social Sciences

## 1 Introduction

The term infants at risk refers to infants with a history that may lead to developmental problems and who exhibit higher‐than‐average mortality and morbidity rates (Formiga et al. 2018). These infants may experience developmental delays due to maternal factors as well as complications arising during the prenatal, natal, and postnatal periods. Considering the importance of motor ability for overall child development, delays or impairments in motor performance may lead to deficiencies across multiple domains extending beyond motor skills alone. Delayed motor skills or abnormal motor performance during infancy may indicate the presence of a motor developmental disorder or other developmental problems. Although these signs may be nonspecific, early identification is critical to initiate interventions that can improve outcomes. Timely intervention can prevent or minimize developmental delays. In addition, secondary complications such as muscle weakness and contractures may be prevented through early intervention. In this study, the term “at‐risk infant” refers to infants with a history of perinatal stroke, perinatal asphyxia, hypoxic‐ischemic encephalopathy (HIE), germinal matrix/intraventricular hemorrhage (GMH/IVH), periventricular leukomalacia (PVL) or a gestational age of ≤32 weeks. These infants are considered to be at risk of neurodevelopmental delays (Morgan et al. 2021; Orton et al. 1996; Einspieler et al. 1997).

In this context, early intervention encompasses multidisciplinary services provided from birth to 3 years of age. These services aim to promote the health and well‐being of infants at risk for developmental disorders, minimize developmental delays by supporting skills across multiple domains, prevent functional impairments, facilitate the reduction of existing limitations, and enhance overall family functioning (Blackman 2002). Early intervention approaches have been implemented in many countries for decades to improve developmental outcomes in infants at risk and to enhance family well‐being. In Türkiye, services for infants with special needs and families are delivered in center‐based settings under the supervision of a physiotherapist. However, early intervention approaches implemented in natural environments support child development more effectively than traditional intervention models delivered in clinical or center‐based settings (Adolph and Kretch 2015; Flôres et al. 2019; Gibson 1988). Natural environments provide contexts in which children learn behaviors identified as necessary by adults and support child‐initiated activities within daily routines. In addition, collaboration with the family increases parental involvement and positively influences intervention outcomes (Morgan et al. 2014; Hamer et al. 2017; Hielkema et al. 2010). Improvements in functional task performance and child developmental gains are observed, while parental satisfaction with health services increases and psychological well‐being is positively affected. In recent years, the importance of early intervention approaches implemented with active family participation has gained increasing attention in the literature. The quality of motor practices provided by caregivers and parents, as well as within the home environment and educational settings, critically influences all aspects of development (Howes et al. 2008; Krauss 2000; Pianta et al. 2008). Activity‐based early interventions are approaches based on the child's active participation in meaningful and goal‐oriented activities within their daily life. Rather than therapist‐directed repetitive exercises, these approaches aim to promote the repetition of functional activities in a natural and enriched environment, and to improve motor learning and developmental gains through collaboration with the family (Øberg et al. 2023).

The SAFE (S—Sensory Strategies, A—Activity‐Based Motor Training, F—Family Collaboration, E—Environmental Enrichment) early intervention approach has recently been introduced into the literature as a novel early intervention model developed in Turkey (Yildiz et al. 2025; Yildiz et al. 2025; Apaydın et al. 2023). The SAFE early intervention approach aims to implement activity‐supported sensorimotor experiences for the infant or child beginning in early life, delivered in natural environments through family collaboration. By capitalizing on the period in which neural plasticity is most pronounced, this approach seeks to provide comprehensive support grounded in interaction and communication across psychomotor, cognitive, social, emotional, and language domains. Activity frequency is increased in accordance with a structured guide developed to ensure accurate parental implementation of the intervention. The study was designed to monitor developmental outcomes at 2 and 3 years of age in infants who received the SAFE early intervention approach and to compare these outcomes with those of infants who received Neurodevelopmental Treatment (NDT).

Previous studies have reported that the SAFE early intervention approach applied to infants at risk across different age groups resulted in greater improvements across various developmental domains compared with the NDT approach and was found to be reliable (Yildiz et al. 2025; Yildiz et al. 2025; Apaydın et al. 2023). A large proportion of the studies in the literature have focused on the short‐term effects of early intervention approaches, and evidence regarding the long‐term outcomes—specifically whether these effects persist into early childhood—remains limited (McGlade et al. 2023). Although studies reporting long‐term follow‐up outcomes of various early intervention approaches are available in the literature, long‐term follow‐up results of the SAFE early intervention approach have not yet been demonstrated. The aim of this study was to examine the long‐term effects of the SAFE early intervention approach across all developmental domains in infants who received this intervention and, based on the findings, to guide the continuation of developmental support.

## 2 Materials and Methods

### 2.1 Study Setting and Timeframe

The study was conducted between September 2022 and March 2025 at the Developmental Physiotherapy and Pediatric Rehabilitation Unit of the Faculty of Health Sciences at Gazi University. The study was approved by the Ethics Commission of Gazi University with the research code number 2025‐1508.

### 2.2 Participants

This study was designed as a 3‐year follow‐up to a doctoral thesis evaluating the effectiveness of the SAFE Early Intervention Approach (Yildiz et al. 2025). The children included in our study are those who previously took part in the published randomized controlled SAFE trial. In this study, the participants’ baseline demographic, clinical and developmental characteristics were assessed, and no significant difference was found between the SAFE and NDT groups before the intervention (Yildiz et al. 2025).

A total of 41 at‐risk infants had been included in the initial study. As part of this study, all families who had participated in the initial study were contacted, and the children of those families who could be reached and agreed to take part in the follow‐up study were included in the assessment. The final sample comprised a total of 23 children: 13 in the SAFE group and 10 in the NDT group. During the follow‐up period, three children were unable to take part in the Bayley‐III assessment in the third year due to being diagnosed with cerebral palsy. Consequently, missing data arose in the third‐year Bayley‐III assessment, and these data were addressed in the statistical analyses using the multiple imputation method.

The study included infants with a corrected age of 3 months who were at neurodevelopmental risk. Neurodevelopmental risk was defined as perinatal stroke, perinatal asphyxia, hypoxic‐ischemic encephalopathy (HIE), germinal matrix hemorrhage/intraventricular hemorrhage (GMH/IVH), periventricular leukomalacia (PVL) or being born at ≤32 weeks’ gestation. The exclusion criteria for the study were the presence of a congenital anomaly or genetic syndrome (e.g., Down's syndrome) and at least one parent not speaking Turkish. The demographic and clinical characteristics of the participants, together with the baseline comparisons between the two groups, are presented in Table 1.

**TABLE 1:** Demographic and clinical characteristics of the infants and baseline comparison between the SAFE and NDT groups.

Characteristics	SAFE (*n* = 13)	NDT (*n* = 10)	*p*
**Age at second‐year assessment (months)**	23.75 (23.50/24.75)	24.25 (23.75/24.25)	0.473
**Age at third‐year assessment (months)**	36.75 (35.50/36.75)	36.50 (35.50/36.75)	0.356
**Gestational age (weeks)**	32.23 ± 5.10	34.90 ± 3.45	0.170
**Birth weight (g)**	1868.46 ± 831.16	2377.50 ± 997.14	0.196
**NICU stay (days)**	40 (6/95)	27.50 (7/72)	0.514
**Sex**			
Female	6 (46.15)	6 (60)	0.680
Male	7 (53.85)	4 (40)	
**Preterm birth**			
Yes	10 (76.92)	7 (70)	1.000
No	3 (23.08)	3 (30)	
**Type of conception**			
Spontaneous	7 (53.85)	6 (60)	1.000
IVF	6 (46.15)	4 (40)	
**Mode of delivery**			
Vaginal	2 (15.38)	2 (20)	1.000
Caesarean	11 (84.62)	8 (80)	
**Multiple pregnancy**			
Yes	5 (38.46)	3 (30)	1.000
No	8 (61.54)	7 (70)	
**Epilepsy**			
Yes	2 (15.38)	0 (0)	0.486
No	11 (84.62)	10 (100)	
**Maternal education (years)**			
< 12	3 (23.08)	1 (10)	
= 12	4 (30.77)	4 (40)	0.701
> 12	6 (46.15)	5 (50)	
**Paternal education (years)**			
< 12	2 (15.38)	0 (0)	
= 12	3 (23.08)	6 (60)	0.135
> 12	8 (61.54)	4 (40)	

*Note*: Data are presented as mean ± SD, median (minimum/maximum) or *n* (%). Continuous variables reported as mean ± SD were compared using the independent‐samples *t*‐test; those reported as median (minimum/maximum) were compared using the Mann–Whitney *U* test; categorical variables reported as *n* (%) were compared using the chi‐square or Fisher's exact test.

Abbreviations: IVF, in vitro fertilization; NDT, neurodevelopmental treatment; NICU, neonatal intensive care unit; SAFE, S—Sensory Strategies, A—Activity Based Motor Training, F—Family Collaboration, E—Environmental Enrichment; SD, standard deviation; T1, first assessment (≈12 months, 1 year); T2, second assessment (≈24 months, 2 years); T3, third assessment (≈36 months, 3 years).

### 2.3 Intervention

#### 2.3.1 SAFE Early Intervention Approach

The SAFE early intervention approach is a family‐centered, activity‐focused program based on the use of sensory strategies, planned in line with the baby's individual needs and the family's priorities and goals. Activity‐focused interventions were embedded within meaningful daily routines to encourage active participation of both the infant and the family. The intervention is supported by a coaching approach designed to take place in a natural and enriched environment, with the family playing an active role in the process. Monthly home visits were carried out throughout the intervention; during these visits, parents were given advice on how to support their baby's development within daily routines, the home environment was assessed, and necessary environmental adjustments were made. Throughout the intervention, as part of activity‐based training, the program focused on supporting voluntary movements, integrating sensory inputs (tactile, vestibular, visual, auditory and oral) into daily activities, and incorporating practices that support communication, social interaction and parent‐infant interaction. (Yildiz et al. 2025; Yildiz et al. 2025; Apaydın et al. 2023; McGlade et al. 2023; Yildiz et al. 2024)

#### 2.3.2 NDT Approach

The NDT‐based early intervention program was planned to target the development of motor skills appropriate to the child's developmental level. Parents were taught therapeutic holds and facilitation techniques through a home program in a practical, hands‐on manner. The implementation of the program was monitored through weekly telephone consultations; infants were called in for clinical assessments each month, and the program was updated according to the infant's needs. Interventions focused on improving postural control, facilitating voluntary movements, weight shifting, reaching, rolling, sitting and normal movement patterns (Yildiz et al. 2024; Howle 2002)

#### 2.3.3 Data Collection Instruments

The 2‐ and 3‐year follow‐up assessments were carried out by a pediatric physiotherapist specializing in the field, who was independent of the physiotherapist implementing the intervention approach and was blind to the participants’ group allocations. During the assessment phase, semi‐structured interviews were conducted with all parents to identify the children's daily routines and the families’ priority intervention goals. Information regarding demographic characteristics, birth history, and the presence of any diagnoses was obtained from parents through face‐to‐face interviews. The information obtained from these interviews was integrated with the assessment findings and used to revise and develop individualized intervention plans. Motor development was evaluated using the Bayley Scales of Infant and Toddler Development, Third Edition (BAYLEY‐III), and sensory development was assessed using Dunn's Sensory Profile (7–36 months).

##### 2.3.3.1 Bayley Scales of Infant and Toddler Development, Third Edition (Bayley‐III)

The Bayley Scales of Infant and Toddler Development, Third Edition (Bayley‐III) is an assessment tool that evaluates five developmental domains in children aged 1–42 months. These domains include cognitive development; language development (receptive and expressive language skills); motor development (gross and fine motor skills); social‐emotional development; and adaptive behavior. In the present study, the Bayley‐III was used to assess cognitive, motor, and language development. Although the scale does not provide an overall total score, it yields separate raw and scaled scores for each domain, as well as composite scores and percentile ranks for each subscale. All items are scored as 0 (unable to perform the required skill) or 1 (able to perform the required skill), and a raw score is calculated. Raw scores are first converted into scaled scores and subsequently into composite scores (mean: 100; standard deviation: 15). A composite score below 85 indicates developmental delay in the corresponding domain. The composite scores of the Bayley‐III assessment were evaluated and interpreted with reference to the scale's original normative scores (Elbasan et al. 2017).

##### 2.3.3.2 Dunn's Sensory Profile—Caregiver Questionnaire (7–36 Months)

Sensory development was evaluated using Dunn's Sensory Profile—Caregiver Questionnaire (7–36 months). The questionnaire was completed by the parent who provided primary care. The instrument consists of 125 items across 14 sensory processing subdomains. These items provide assessment in the domains of Sensory Processing (6 subdomains), Sensory Modulation (5 subdomains), and Behavioral and Emotional Responses (3 subdomains) (Bayley 2006). Items are rated on a five‐point Likert scale. Lower total scores indicate greater difficulties in sensory processing. Subscale scores are interpreted according to normative data as “typical performance,” “probable difference,” or “definite difference.” The instrument is a standardized assessment tool with established applicability for evaluating sensory processing in children. In the Sensory Profile assessments, the version of the scale for which Turkish validity and reliability had been established was used; scoring and interpretation were carried out in accordance with the scale's standard administration guidelines (Dunn 1999).

### 2.4 Statistical Analysis

Statistical analyses were performed using IBM SPSS Statistics 30.0 (SPSS Inc., Chicago, IL, USA). The distribution characteristics of continuous variables were assessed using both visual and analytical methods; in this context, histograms and normal probability plots were examined, and the Shapiro–Wilk test was used to assess normality, accounting for sample size. Descriptive statistics were presented as the mean and standard deviation, or the median and minimum and maximum values, for continuous variables, depending on their distribution characteristics, while categorical variables were presented as frequencies and percentages.

When examining the baseline demographic and clinical characteristics of the groups, the independent samples *t*‐test was used for continuously distributed variables that followed a normal distribution, while the Mann–Whitney *U* test was used for continuously distributed variables that did not follow a normal distribution; for comparisons of categorical variables between groups, the chi‐squared test was applied, and Fisher's exact test was used where necessary (Kayihan et al. 2015).

The nature of the missing data pattern was examined in detail before proceeding to the main analyses. While complete Bayley‐III composite scores were obtained for all participants in the second‐year assessment, it was determined that were missing for three infants in each developmental domain in the third‐year assessment. The Little's MCAR test was applied to determine whether the missing data were random (χ^2^ = 5.743; df = 8; *p* = 0.676), and the assumption that the data were completely missing at random could not be rejected. Based on this finding, the multiple imputation method was chosen to handle missing observations, and 20 complete data sets were generated (Barton and Peat 2014).

To compare the developmental patterns of the groups between the ages of two and three, a two‐way repeated‐measures analysis of variance was used for each Bayley‐III domain. This analysis was carried out separately for each of the 20 data sets created, and the estimates obtained were pooled using Rubin's pooling rules, which account for both within‐ and between‐allocation variability, to arrive at the final results. In this two‐time‐point design, the reduction of the time × group interaction to a single degree of freedom eliminated the well‐known difficulties associated with pooling across multiple degrees of freedom, thereby enabling the direct and consistent pooling of the interaction effect. Within‐group variations and between‐group differences at each time point were analyzed as simple effects, with a Bonferroni correction applied to control for multiple comparisons (Little et al. 2012).

Due to the high proportion of missing data observed in the analysis of sensory profile follow‐up data, a cross‐sectional analysis was conducted using the available data. This approach avoided longitudinal analyses, which could have increased bias due to missing data. In comparisons between groups during the second‐ and third‐year assessments, either an independent‐samples *t*‐test or a Mann–Whitney *U* test was used, depending on the distributions of the continuous variables.

Effect sizes were interpreted using Cohen's *d* for within‐group changes and partial eta‐squared for the time × group interaction; For Cohen's *d*, values of 0.20, 0.50 and 0.80 were taken as criteria for small, medium, and large effects, respectively, while for partial chi‐square, values of 0.01, 0.06 and 0.14 were used as criteria for small, medium, and large effects, respectively. In all analyses, a *p*‐value of less than 0.05 was taken as the threshold for statistical significance (Rubin 1987).

## 3 Results

The study included 23 infants: 13 in the SAFE group and 10 in the NDT group. When the two groups were compared at baseline, there was no difference between the groups in corrected age at the second‐ and third‐year assessments, gestational age, birth weight, or duration of stay in the neonatal intensive care unit (*p* > 0.05). Likewise, no differences were observed for any of the categorical variables, including sex, preterm birth, type of conception, mode of delivery, multiple pregnancy, presence of epilepsy, or maternal and paternal education level (*p* > 0.05) between groups.

Of the twenty‐three infants included in the study, third‐year cognitive assessment data were unavailable for three infants, whereas complete data were available for all participants at the second‐year assessment; the missing observations were therefore addressed through multiple imputation, and the pooled estimates are reported below.

With respect to the within‐group comparisons of the Bayley‐III cognitive composite score, a statistically significant improvement was observed from the second to the third year in both groups, reaching significance in the SAFE group (*p^a^* = 0.002) as well as in the NDT group (*p^a^* = 0.012), with large within‐group effect sizes in each group (Cohen's *d* = 0.970 and *d* = 0.850, respectively). When the groups were compared, no significant difference was detected at either the second‐year (*p^b^* = 0.312) or the third‐year assessment (*p^b^* = 0.133). The time × group interaction did not reach statistical significance (*p^c^* = 0.540; *η* ^2^ *_p_* = 0.023) (Table 2).

**TABLE 2:** Bayley‐III composite scores of the SAFE and NDT groups at the second‐ and third‐year assessments.

Outcome	Group	Age 2 years Mean ± SE	Age 3 years Mean ± SE	Change Mean ± SE	*p^a^*	Cohen's *d* (95% CI)	Time × group		
							*F*	*p^c^*	*η* ^2^ *_p_* (95% CI)
Bayley‐III cognitive composite score	SAFE	93.85 ± 3.80	106.84 ± 5.57	12.99 ± 4.19	**0.002**	0.970 (0.310, 1.630)	0.376	0.540	0.023 (0.000, 0.253)
	NDT	102.00 ± 7.12	120.12 ± 6.80	18.12 ± 7.19	**0.012**	0.850 (0.120, 1.570)			
	***p^b^***	0.312	0.133						
Bayley‐III language composite score	SAFE	98.31 ± 4.46	116.30 ± 6.14	17.99 ± 5.34	**0.001**	1.090 (0.400, 1.780)	0.308	0.579	0.053 (0.000, 0.307)
	NDT	86.20 ± 8.02	109.56 ± 6.44	23.36 ± 7.65	**0.002**	1.010 (0.250, 1.780)			
	***p^b^***	0.187	0.466						
Bayley‐III motor composite score	SAFE	93.62 ± 2.49	97.17 ± 4.84	3.55 ± 5.35	0.507	0.230 (−0.320, 0.780)	0.036	0.851	0.001 (0.000, 0.137)
	NDT	83.70 ± 6.59	85.20 ± 7.63	1.50 ± 9.10	0.869	0.060 (−0.560, 0.680)			
	***p^b^***	0.159	0.205						

*Note*: Data are presented as estimated marginal means ± standard error, pooled across 20 imputed datasets using Rubin's rules. Within‐group change from the second to the third year (*p^a^*) and between‐group differences at each assessment (*p^b^*) were obtained as simple effects with Bonferroni correction for multiple comparisons; the time × group interaction (*p^c^*) was derived from a two‐way repeated‐measures analysis of variance conducted within each imputed dataset and pooled across imputations. Cohen's *d* and partial eta‐squared are reported with their 95% confidence intervals.

Abbreviations: Bayley‐III, Bayley Scales of Infant and Toddler Development, Third Edition; CI, confidence interval; NDT, neurodevelopmental treatment; SAFE, S—Sensory Strategies, A—Activity Based Motor Training, F—Family Collaboration, E—Environmental Enrichment; SE, standard error; *η* ^2^ *_p_*: partial eta‐squared.

Bold values indicate statistically significant results (*p* < 0.05).

Regarding the within‐group analyses of the Bayley‐III language composite score, a significant gain was identified between the second and third years in both study groups, attaining significance in the SAFE group (*p^a^* = 0.001) and likewise in the NDT group (*p^a^* = 0.002), accompanied by large within‐group effect sizes in each group (Cohen's *d* = 1.090 and *d* = 1.010, respectively). Upon comparison between the two groups, no significant difference emerged at either the second‐year (*p^b^* = 0.187) or the third‐year (*p^b^* = 0.466) assessment. The time × group interaction failed to attain statistical significance (*p^c^* = 0.579; *η* ^2^ *_p_* = 0.053) (Table 2).

Concerning the within‐group evaluations of the Bayley‐III motor composite score, and in contrast to the pattern observed in the two preceding domains, no significant change was detected from the second to the third year in either group, with the difference remaining non‐significant in the SAFE group (*p^a^* = 0.507) as well as in the NDT group (*p^a^* = 0.869); accordingly, the within‐group effect sizes were small in each group (Cohen's *d* = 0.230 and *d* = 0.060, respectively). When the two groups were compared, no significant difference was found at either the second‐year (*p^b^* = 0.159) or the third‐year assessment (*p^b^* = 0.205). The time × group interaction proved to be the weakest among the three domains and did not attain statistical significance (*p^c^* = 0.851; *η* ^2^ *_p_* = 0.001) (Table 2).

Given that a significant proportion of the follow‐up data was missing from the sensory profile assessments, longitudinal analyses were considered inappropriate for these results. Consequently, the sensory profile scores were analyzed cross‐sectionally at each assessment time point using the available data.

Cross‐sectional comparisons of the Sensory Profile scores at 2 years of age are presented in Table 3. Significant between‐group differences were observed in the General Processing, Sensory Sensitivity, and Sensory Avoiding subscales, with higher performance in the SAFE group (*p* < 0.05).

**TABLE 3:** Comparison of sensory development in children.

**Dunn's sensory profile**	General processing	10 (91–12)	16 (14–18)	**0.005**	10 (8–12)	12 (9–13)	0.279
	Auditory	41 (35–45)	36 (35–42)	0.429	35 (34–38)	36 (35–42)	0.277
	Visual	24 (20–27)	20 (18–26)	0.237	25 (22–26)	26 (22–28)	0.308
	Tactile	46 (40–54)	49 (44–57)	0.433	47 (43–51)	53 (48–54)	0.099
	Vestibular	18 (17–22)	21 (20–23)	0.130	20 (18–22)	18 (16–22)	0.515
	Oral	30 (25–31)	30 (28–33)	0.644	32 (25–34)	31 (27–31)	0.885
	Low registration	50 (48–53)	50 (48–53)	0.793	48 (45–49)	53 (50–55)	**0.012**
	Sensory seeking	34 (26–43)	30 (22–39)	0.359	34 (31–37)	33 (30–34)	0.720
	Sensory sensitivity	38 (33–42)	48 (43–50)	**0.030**	39 (36–41)	43 (840–45)	0.173
	Sensory avoiding	46 (43–50)	53 (50–57)	**0.036**	41 (37–48)	49 (45–51)	**0.044**

*Note*: Bold values indicate that statistical significance was accepted at *p* < 0.05.

Abbreviations: SAFE, S—Sensory Strategies, A—Activity Based Motor Training, F—Family Collaboration, E—Environmental Enrichment, NDT: neurodevelopmental treatment.

Cross‐sectional comparisons of the Sensory Profile scores at 3 years of age are also presented in Table 3. At this assessment, significant between‐group differences remained only for the Low Registration and Sensory Avoiding quadrants in favor of the SAFE group (*p* < 0.05). No significant between‐group differences were observed for the remaining Sensory Profile domains (*p* > 0.05).

**FIGURE 1:**
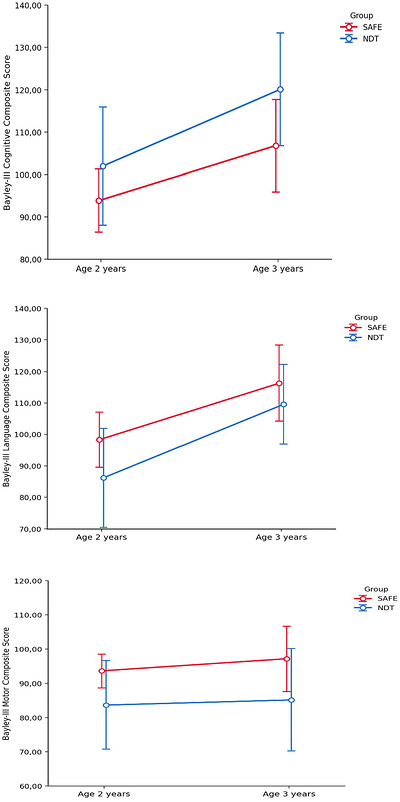
Mean Bayley‐III cognitive (A), language (B), and motor (C) composite score trajectories of the SAFE and NDT groups across the second‐ and third‐year assessments. Data points represent estimated marginal means pooled across 20 imputed datasets using Rubin's rules, and error bars denote the corresponding 95% confidence intervals. The red lines represent the SAFE group and the blue lines represent the NDT group.

The estimated marginal means and their 95% confidence intervals for both groups across the two assessment points are presented in Figure 1 for the cognitive (A), language (B), and motor (C) composite scores.

Among the 22 children assessed at 2 years of age, 9 (40.9%) were male, and 13 (59.1%) were female. Among the 19 children assessed at 3 years of age, 9 (47.36%) were male and 10 (52.64%) were female. Gestational age, sex, treatment group allocation, and related data are presented in Table 1.

Comparative analyses of cognitive, gross motor, fine motor, expressive language, receptive language, and sensory development scores at 2 years of age are presented in Table 2. The comparison revealed a significant difference in fine motor development in favor of the SAFE group (*p* < 0.05). In the Sensory Profile, significant differences were also identified in the general processing, sensory sensitivity, and sensory avoiding subscale scores in favor of the SAFE group (*p* < 0.05).

Comparative analyses of cognitive, gross motor, fine motor, expressive language, receptive language, and sensory development scores at 3 years of age are presented in Table 2. At 3 years of age, the difference between groups in motor development was no longer observed. However, in the Sensory Profile, significant differences were identified in the low registration and sensory avoiding quadrant scores in favor of the SAFE group (*p* < 0.05).

## 4 Discussion

Extensive research on early intervention during infancy and childhood has been conducted in recent years, and short‐term outcomes have been widely reported. However, studies evaluating the long‐term effects of these interventions remain limited (Cohen 2013; Novak et al. 2013; Novak et al. 2020). The results of the longitudinal analysis showed significant progress in cognitive and language development in both groups from age 2 to 3, but no significant change in motor development. Furthermore, no significant time × group interaction was observed for developmental change. In cross‐sectional sensory profile assessments, however, the SAFE group showed better performance in certain sensory domains. These findings suggest that while both early intervention approaches facilitate developmental progress, the SAFE approach may facilitate greater development, particularly in specific sensory domains. However, due to the limited sample size in the sensory development analyses, these findings must be interpreted with caution.

These findings are consistent with previous research in the literature demonstrating the effects of early intervention on children's development. In particular, various studies and systematic reviews have reported that early intervention approaches centered on family collaboration, activity‐based, and implemented in natural settings have positive effects on motor, cognitive, and language development. However, while most of these studies have focused on short‐term outcomes, there are limited long‐term follow‐up studies examining the sustainability of intervention effectiveness through to the pre‐school period. Therefore, our study contributes to the existing literature by providing long‐term follow‐up data (Orton et al. 2024)

In the present study, a significant change in motor composite scores was observed in both the SAFE and NDT groups during the period between the ages of two and three; however, no significant time × group interaction was observed. This finding suggests that, in the early years, both intervention approaches may have produced similar long‐term effects on motor development. However, the finding of a significant difference in favor of the SAFE group in fine motor skills at the 2‐year assessment may be related to the program's activity‐based structure, which integrates sensory strategies (Yildiz et al. 2025). In the SAFE approach, integrating sensory‐motor experiences into daily living activities within a natural and enriched environment, in collaboration with families, may have increased the repetition of fine motor skills, such as hand‐eye coordination, object manipulation, and coordinated use of both hands. This finding is consistent with the results reported by Morgan et al. in their study evaluating the GAME early intervention approach, which indicated that goal‐oriented and activity‐based interventions carried out in a natural environment support the acquisition and development of motor skills (Morgan et al. 2014).

Preterm infants are more likely than full‐term peers to exhibit sensory processing difficulties around 1 year of age, which may adversely affect motor skills. These findings support the association between sensory processing and motor performance in early childhood (McGlade et al. 2023; Dusing et al. 2015). It is thought that early interventions—including sensory strategies and those that support sensory development—may have positive effects on developmental outcomes. Although our study did not identify significant differences in the groups’ developmental patterns in the longitudinal analyses of Bayley‐III motor composite scores, differences favored the SAFE group were observed in certain sensory domains in the cross‐sectional sensory development assessments. In particular, positive developments in favor of the SAFE group were observed in the areas of general sensory processing, sensory sensitivity, and sensory avoidance at the 2‐year assessment; while at the 3‐year assessment, positive developments were observed in the areas of low registration and sensory avoidance. These findings suggest that the SAFE approach may be particularly effective in supporting specific subdimensions of sensory domains. Given the reciprocal relationship between sensory and motor development, it can be anticipated that differences in sensory development domains may positively impact children's performance in daily life. These findings suggest that early interventions supporting sensory processing skills can enhance not only sensory processing proficiencies but also children's participation in daily activities and functional independence (Wickremasinghe et al. 2013). However, the limited sample size due to missing follow‐up data in the sensory assessments necessitates that these findings be carefully validated in future studies with larger samples.

This finding is consistent with previous studies indicating that sensory processing skills serve as an indirect yet powerful determinant of motor performance and participation (Celik et al. 2018). In particular, the study by Yildiz et al. (2025), which demonstrated an association between sensory processing and motor skills in 12‐month‐old infants, supports the influence of early sensory‐based approaches on motor outcomes. Similarly, a randomized controlled study examining the early effects of the SAFE approach reported greater improvements across developmental domains in infants who received the SAFE intervention (Yildiz et al. 2025). The present study suggests that certain developmental milestones—such as specific areas of sensory development identified at an early stage using the SAFE approach—may also be observed in assessments at ages 2 and 3.

Christakis, DA concluded that a multifaceted clinic‐based approach designed to promote parent–child interactions shows promising potential (Christakis et al. 2019). In this study, no difference was found in language development between the groups across the longitudinal analyses. However, the family‐centered structure of the SAFE approach and its support for parent‐child interaction within everyday activities may have indirectly supported language development. Although no significant difference was found between the groups, the observed significant improvement in language composite scores in both groups from age 2 to 3 supports the positive effects of early intervention programs on communication and language development.

This study has several limitations. The small sample size and participant attrition at 3 years of age limit the generalizability of the findings. Missing data from the Bayley‐III assessments at age three were imputed using multiple imputation. However, because sufficient matched data could not be obtained for the sensory assessments due to loss to follow‐up, the sensory profile follow‐up results could be evaluated only cross‐sectionally. Consequently, the findings relating to the sensory profile need to be validated using larger samples and longitudinal analyses. Although the study was not designed as a randomized long‐term follow‐up trial, it represents a continuation of an initial doctoral dissertation. In addition, reliance on parent‐reported measures for sensory processing assessment may introduce potential reporting bias. However, the fact that all evaluations were conducted by a single experienced physiotherapist who was independent of the physiotherapist administering the intervention and was blind to group allocation may be considered a strength in terms of measurement consistency.

In conclusion, this study is one of the first to conduct a longitudinal analysis of the 2‐ and 3‐year follow‐up outcomes of the SAFE early intervention approach. The findings reveal that children receiving both the SAFE and NDT approaches showed significant progress over time in cognitive and language development; however, the developmental patterns of the groups were similar. The observation of differences in favor of the SAFE group in certain areas of sensory development in cross‐sectional analyses suggests that this approach may offer potential benefits, particularly in supporting sensory development. Further evaluation of SAFE—a unique early intervention approach developed in Türkiye, centered on the family and based on sensory strategies and activity‐focused motor training—through studies involving larger samples and longer follow‐up periods will contribute to a better understanding of its long‐term developmental effects.

## Author Contributions

BE: development of the theoretical and practical framework, study design, literature review, manuscript drafting, and critical revision. EYÇ: literature review, data collection, and manuscript drafting. PAE: Literature review, data collection, and manuscript drafting. KG: Conceptualization.

## Funding

The authors have nothing to report.

## Ethics Statement

Ethical approval for this study was obtained from the Ethics Committee of Gazi University (Approval No: 2025‐1508). The study was conducted in accordance with the ethical principles of the Declaration of Helsinki.

## Conflicts of Interest

The authors declare that they have no known conflicts of interest or personal relationships that could have influenced the study reported in this article.

## Data Availability

The datasets generated and/or analyzed during the current study are available from the corresponding author upon reasonable request.
